# Assessing the value for money of a registry: an exploratory analysis of the Dutch Arthroplasty Register

**DOI:** 10.2340/17453674.2026.45553

**Published:** 2026-03-10

**Authors:** Mirthe H W VAN VEGHEL, Gerjon HANNINK, B Willem SCHREURS, Janneke P C GRUTTERS

**Affiliations:** 1 Department of Orthopaedics, Radboud University Medical Center, Nijmegen; 2 Department of Medical Imaging, Radboud University Medical Center, Nijmegen; 3 IQ Health Science Department, Radboud University Medical Center, Nijmegen, the Netherlands

## Abstract

**Background and purpose:**

It is unclear whether arthroplasty registries provide value for money. We aimed to provide insight into this value for money by relating the costs of the Dutch Arthroplasty Register (LROI) to potential benefits from preventing revisions.

**Methods:**

We included all primary total hip (n = 276,252) and knee (n = 217,901) arthroplasties (THA/TKA), and all first revisions of these THAs (n = 8,604) and TKAs (n = 8,745) from the LROI between 2014 and 2022. Threshold analyses estimated at which number of prevented revisions the benefits (i.e., savings and increased quality of life) outweighed the LROI costs. Scenario analyses explored whether the required revision reductions were feasible, including the minimum revision reduction required per hospital, revision reduction at outlier hospitals only, and the potential impact of temporal changes in national 1-year revision rates.

**Results:**

The LROI costs were €5,697,120 for THA registration and €4,532,920 for TKA registration between 2014 and 2022. Preventing 119 to 145 THA revisions and 106 to 134 TKA revisions would be required in the current patient population over 30 years for the benefits to outweigh these LROI costs between 2014 and 2022, depending on revision costs. Each Dutch hospital should prevent 1.2 to 1.5 THA revisions and 1.1 to 1.3 TKA revisions to achieve the required revision reduction. Moreover, 306 THA revisions and 140 TKA revisions could be prevented if outlier hospitals improved their 1-year overall revision rate to the upper 99% control limit. Compared with the calendar years showing the highest revision rates, applying 2022 1-year revision rates to 2023 procedure volumes resulted in reductions of 1.5% for THAs and 0.3% for TKAs.

**Conclusion:**

Preventing approximately 1–2 revisions per hospital over 30 years would offset the LROI’s registration costs. Although causality cannot be established, these findings suggest that national arthroplasty registries may provide good value for money when actively used to reduce revision rates.

Total hip and knee arthroplasty (THA/TKA) are among the most cost-effective surgical interventions, relieving pain, improving joint function, and enhancing quality of life for patients with advanced joint diseases [1,2]. Since 1969, several jurisdictions have established arthroplasty registries to monitor and evaluate outcomes after arthroplasty [3-5]. These registries contain real-world data on patient, prosthesis, and procedure characteristics, enabling case identification in product recalls or when patient identification is needed [6].

Effective use of arthroplasty registries may improve health outcomes and decrease healthcare costs, as identifying factors related to adverse outcomes, such as higher revision rates, may reduce morbidity, mortality, and costs associated with subsequent surgeries [3,6]. However, establishing and maintaining these registries is costly. Limited evidence exists on whether they provide value for money due to challenges in developing a rigorous study design, including the absence of a suitable control group [7]. An economic evaluation of the Australian Orthopaedic Association National Joint Replacement Registry measured the reduction in revision burden in hip and knee arthroplasties since its inception [8]. The estimated net benefit was $53 million, with changes in revision burden attributed to the registry. However, quantifying the impact of registries on revision burden over time is complex, as improvements may result from other trends, such as improved surgical protocols [7,9].

Beyond clinical and economic outcomes, national arthroplasty registries are quality databases contributing to transparency and accountability in healthcare. By systematically collecting and reporting real-world data, registries strengthen public trust in providers and the medical device industry and serve as essential instruments for post-market surveillance [10]. They support regulatory oversight and provide a foundation for evidence-based evaluation of implant performance and quality improvement.

Understanding the economic and societal impact of arthroplasty registries is crucial for healthcare decision-makers, policymakers, and other stakeholders, especially considering concerns over healthcare affordability and workforce shortages. Because these registries require significant investments of money and time, their benefits should outweigh the costs. Despite the challenges of developing a rigorous study design without a control group, understanding the added value of an arthroplasty registry remains important [7]. Therefore, we aimed to provide insight into the value for money by relating the costs of the Dutch Arthroplasty Register (LROI) to potential benefits from preventing revisions.

## Methods

A health economic decision model was developed using THA and TKA data from the LROI, as the LROI receives reimbursement per registered THA or TKA from health insurers. The LROI is the national population-based arthroplasty register of the Netherlands, covering 100% of Dutch hospitals with 98% completeness for primary and revision THAs and TKAs [11]. The LROI contains data on patient, prosthesis, and procedure characteristics of primary and revision arthroplasties. This study was reported in accordance with the CHEERS guidelines.

### Participants

We included all primary THAs (n = 276,252) and TKAs (n = 217,901), and all first revisions of these THAs (n = 8,604) and TKAs (n = 8,745) registered in the LROI between 2014 and 2022. Additionally, sub-cohorts of patients with primary THAs (n = 83,908, 30%), primary TKAs (n = 56,122, 26%), revision THAs (n = 871, 10%), and revision TKAs (n = 590, 6.7%) were included if they had EuroQol 5 Dimensions (EQ-5D) index scores available both preoperatively and at 12 months postoperatively.

### Health economic decision model

Decision modeling was used to explore the value for money of the LROI. The value of the registry was considered to be its ability to prevent revisions, in terms of both cost savings and impact on quality of life. A revision was defined as a replacement, removal, or addition of 1 or more prosthetic components [11].

For the economic evaluation, the current practice with the LROI in place was compared with a hypothetical scenario without the registry, assuming no registry-related reduction in revision rates. Because the comparator was defined slightly differently for the economic, threshold, and scenario analyses, detailed descriptions of the comparator used in each analysis are provided in Appendix.

**Figure 1:**
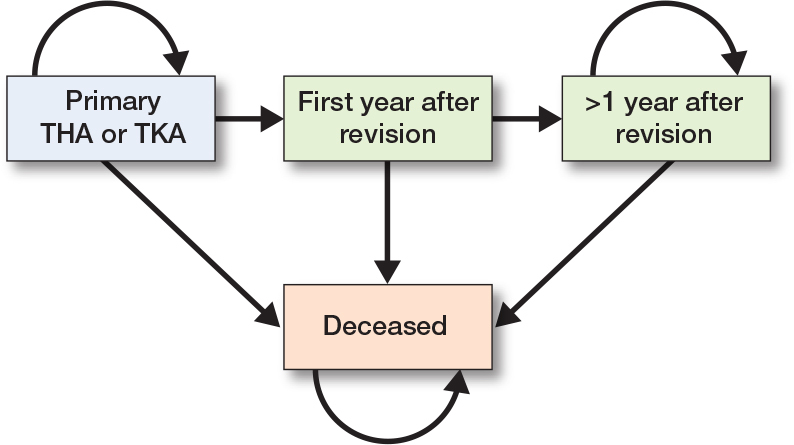
Conceptual overview of patient-level state-transition model. THA = total hip arthroplasty; TKA = total knee arthroplasty.

The costs and quality-adjusted life year (QALY) loss from a revision were estimated using a patient-level state-transition model with 30 yearly cycles, incorporating health states for “Primary THA or TKA,” “First year after revision,” “> 1 year after revision,” and “Deceased” (Figure 1). Tunnel states were created for the revision state (i.e., “First year after revision” and “> 1 year after revision”) to ensure that revision costs were applied only once per patient. A discount rate of 1.5% for effects and 3.0% for costs was applied to each cycle, consistent with the Dutch guideline for health economic evaluations, which recommends discounting health effects at a lower rate than costs because the value society places on good health is expected to increase over time relative to costs [12]. An overview of all model parameters is provided in Table 1, with a detailed description of the model in Appendix.

**Table 1:** Overview of all model parameters

Input parameter	Value	Source
Costs (€)		
Registration of THA or		
TKA in the LROI per		
registered procedure (primary and revision)	20	Zorgverzekeraars Nederland [13]
Revision	15,000; 20,000; 25,000	Klouche et al.; Kallala et al. [15,16]
Transition probabilities		
Mortality	Time-dependent	Statistics Netherlands [25]
Revision	Time-dependent	LROI data
Other		
Discount rate(%) for		
effects	1.5	Zorginstituut Nederland [12]
costs	3.0	Zorginstituut Nederland [12]
Value of a QALY (€)	20,000	Vijgen et al. [14]
Utility scores		
Primary THA	0.83	LROI data
Primary TKA	0.80	LROI data
Revision THA	0.71	LROI data
Revision TKA	0.71	LROI data

THA = total hip arthroplasty; TKA = total knee arthroplasty; LROI = Dutch Arthroplasty Register; QALY = quality-adjusted life year.

### Statistics

The LROI costs were estimated by multiplying the number of registered procedures by the reimbursement rate per procedure (€20) from health insurers [13]. All costs are presented in euros.

Using the health economic model, 2 types of analyses were performed: threshold and scenario analyses. Threshold analyses estimated the number of THA and TKA revisions that needed to be prevented to outweigh the LROI costs from 2014 to 2022. In these threshold analyses, the percentage reduction in revisions was varied in increments of 0.01% to calculate the resulting incremental Net Monetary Benefit (iNMB). The iNMB represents the net monetary value of the additional benefits gained from reducing revisions, taking into account health benefits measured in QALYs, the willingness-to-pay (WTP) for a QALY, and the costs of the revision procedure. A QALY was valued at €20,000, corresponding to the WTP for a QALY for the lowest disease burden in the Netherlands [14].

A positive iNMB indicates that the strategy provides value for money (i.e., the additional benefits outweigh the additional costs), whereas a negative iNMB suggests that it does not. The iNMB was calculated for 3 revision procedure costs (i.e., €15,000, €20,000, and €25,000) [15,16]. Revision reduction percentages were converted into absolute numbers for interpretation. The number of revisions at which the iNMB became positive was determined, indicating the LROI provides value for money, as the benefits outweigh the costs. Uncertainty around the iNMB was not quantified using traditional confidence intervals because it was calculated from deterministic model inputs (revision costs and QALY gains/losses) rather than sample-based estimates. Consequently, the uncertainty does not reflect sampling variability and therefore cannot be described with standard statistical distributions.

Scenario analyses explored whether the required reductions in THA and TKA revisions were considered feasible. The scenario analyses involved 3 scenarios: the minimum reduction in revisions required per hospital, reductions in revisions in outlier hospitals only, and the potential impact of temporal changes in national 1-year revision rates. In the first scenario, the number of hospitals performing primary THAs and TKAs in the Netherlands (2014 to 2020) was assessed. Subsequently, based on the required number of prevented THA and TKA revisions from the threshold analyses, the minimum number of revisions to be prevented per hospital was calculated to meet the total required revision reductions.

For the second scenario, multivariable logistic regression analyses were used to create funnel plots to identify which anonymized hospitals performed significantly worse than others between 2014 and 2022. Outlier hospitals were defined as those with a 1-year overall revision rate, adjusted for sex, age, American Society of Anesthesiologists (ASA) score, diagnosis, body mass index (BMI), smoking status, and Charnley score, exceeding the 99% control limits. It was estimated how many revisions were prevented if outlier hospitals improved their 1-year overall revision rate to the upper 99% control limit. Primary THAs and TKAs were excluded from this analysis if there was a missing value in any of the covariates (THA n = 13,443; TKA n = 10,942) or if they were performed in hospitals with fewer than 50 registered THAs or TKAs between 2014 and 2022 (THA n = 12; TKA n = 97).

For the third scenario, Kaplan–Meier survival analyses were performed to estimate the 1-year overall revision rate for each calendar year between 2014 and 2022. To approximate the potential impact of temporal changes in revision outcomes, we compared the 1-year overall revision rate from the most recent calendar year (i.e., 2022) with the lowest and highest rates observed in earlier years. The resulting differences, representing both potential increases and decreases in revision rates, were then applied to the number of procedures performed in 2023 (THA n = 36,637; TKA n = 28,249) to estimate the corresponding number of additional or reduced revisions.

Uncertainty in the model results was explored through the threshold and scenario analyses already described. Threshold analyses varied the assumed reductions in THA and TKA revisions and the costs of revision procedures to identify the conditions under which the iNMB became positive. Scenario analyses tested the robustness of these findings across different plausible situations, including minimum reductions per hospital, improvements in outlier hospitals, and changes in national revision rates over time. While 95% confidence intervals (CI) were calculated for estimated revision rates, the deterministic threshold and scenario analyses do not have associated confidence intervals.

All results were stratified by THA and TKA. R (version 4.4.0, R Foundation for Statistical Computing, Vienna, Austria) was used to perform all analyses.

### Ethics, data sharing, funding, and disclosures

Data was available from the LROI; however, restrictions apply to the availability of this data, which was used under license for the current study. All data was received completely de-identified. The data used in this study is available upon reasonable request, subject to the approval of the LROI. The LROI uses the opt-out system to require informed consent from patients. This study was funded by the LROI. Complete disclosure of interest forms according to ICMJE are available on the article page, doi: 10.2340/17453674.2026.45553

## Results

### Costs of the LROI

During 2014 and 2022, LROI costs were €10,230,040, with €5,697,120 for THA registration and €4,532,920 for TKA registration. The population characteristics are shown in Table 2.

**Table 2:** Population characteristics of 284,856 total hip arthroplasties and 226,646 total knee arthroplasties performed between 2014 and 2022. Values are count (%) unless otherwise specified

Characteristic	THA n = 284,856	TKA n = 226,646
Female sex	184,196 (65)	143,392 (63)
Missing	147 (0.1)	116 (0.1)
Age, mean (SD)	69 (11)	69 (9)
Type of procedure		
Primary	276,252 (97)	217,901 (96)
Revision	8,604 (3.0)	8,745 (3.9)
Diagnosis		
Osteoarthritis	238,621 (84)	209,275 (92)
No osteoarthritis	36,869 (13)	7,861 (3.5)
Missing	762 (0.3)	765 (0.3)
Not applicable **^a^**	8,604 (3.0)	8,745 (3.9)

a Diagnosis is not recorded for revision procedures in the LROI.

For abbreviations, see Table 1.

### Threshold analyses

**Figure 2:**
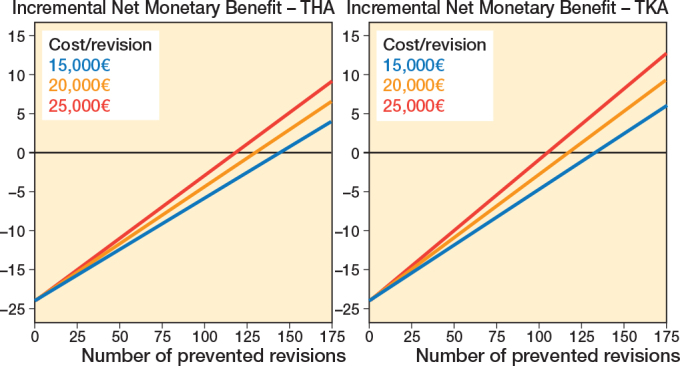
Incremental Net Monetary Benefit of the Dutch Arthroplasty Register (LROI) and the number of revisions that needed to be prevented to outweigh the costs between 2014 and 2022 in the current patient population over 30 years, stratified by total hip arthroplasty (left panel) and total knee arthroplasty (right panel).

Threshold analyses showed that the percentage of THA revisions that needed to be prevented was 0.87% if a revision costs €15,000, 0.78% if a revision costs €20,000, and 0.71% if a revision costs €25,000 for the iNMB to become positive (Table 3). This corresponds to 145, 130, and 119 THA revisions, respectively, that needed to be prevented in the current patient population over 30 years for the LROI to provide value for money between 2014 and 2022 (Figure 2).

**Table 3:** Number of revisions that needed to be prevented in the current patient population over 30 years, based on revision procedure costs and quality of life impact, to outweigh the costs (€) of the Dutch Arthroplasty Register (LROI) between 2014 and 2022, stratified by total hip and knee arthroplasty

Prevented revisions needed	Revision procedure costs		
	€15,000	€20,000	€25,000
Total hip arthroplasty			
% of revisions	0.87	0.78	0.71
Total number	145	130	119
Number per hospital **^a^**	1.5	1.3	1.2
Total knee arthroplasty			
% of revisions	1.47	1.29	1.16
Total number	134	117	106
Number per hospital **^a^**	1.3	1.2	1.1

a 98 hospitals performed total hip arthroplasties between 2014 and 2022, and 100 hospitals performed total knee arthroplasties.

For TKAs, the percentage of revisions that needed to be prevented was 1.47% if a revision costs €15,000, 1.29% if a revision costs €20,000, and 1.16% if a revision costs €25,000 for the iNMB to become positive (see Table 3). This corresponds to 134, 117, and 106 TKA revisions, respectively, that needed to be prevented in the current patient population over 30 years for the LROI to provide value for money between 2014 and 2022 (see Figure 2).

### Scenario analyses

*The first scenario* showed that 98 hospitals performed primary THAs and 100 performed primary TKAs between 2014 and 2022 in the Netherlands. The number of THA revisions each hospital needed to prevent in the current patient population over 30 years was 1.5 if a revision costs €15,000, 1.3 if a revision costs €20,000, and 1.2 if a revision costs €25,000 for the LROI to provide value for money between 2014 and 2022 (see Table 3).

For TKAs, each hospital needed to prevent 1.3 revisions if a revision costs €15,000, 1.2 revisions if a revision costs €20,000, and 1.1 revisions if a revision costs €25,000 in the current patient population over 30 years for the LROI to provide value for money between 2014 and 2022.

**Figure 3:**
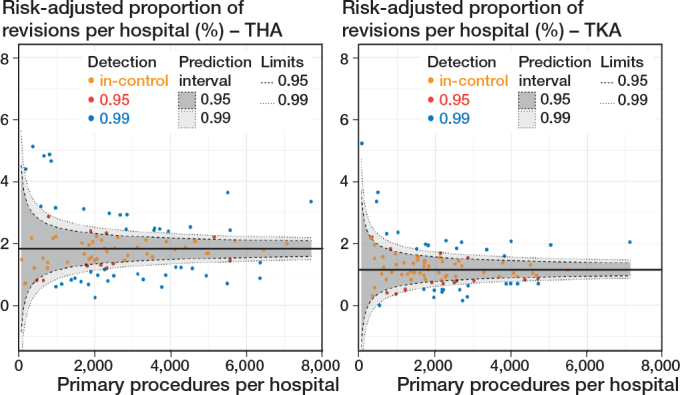
Funnel plot showing the proportion of revisions within 1 year after the primary procedure per hospital between 2014 and 2022, adjusted for sex, age, ASA, diagnosis, BMI, smoking, and Charnley, and stratified by total hip arthroplasty (left panel) and total knee arthroplasty (right panel).

*In the second scenario,* 20 hospitals were outliers for THAs, and 16 for TKAs between 2014 and 2022 (Figure 3). The mean 1-year overall revision rate across all hospitals was 1.82% for THAs and 1.16% for TKAs. Outlier hospitals had 1-year overall revision rates between 2.38% and 5.13% for THAs, and between 1.60% and 5.22% for TKAs. The difference between the 1-year overall revision rate with the upper 99% control limit across outlier hospitals was between 0.05% and 1.95% for THAs and between 0.02% and 1.35% for TKAs. Improving these outlier hospitals to the upper 99% control limit could have prevented 306 THA revisions and 140 TKA revisions between 2014 and 2022.

**Figure 4:**
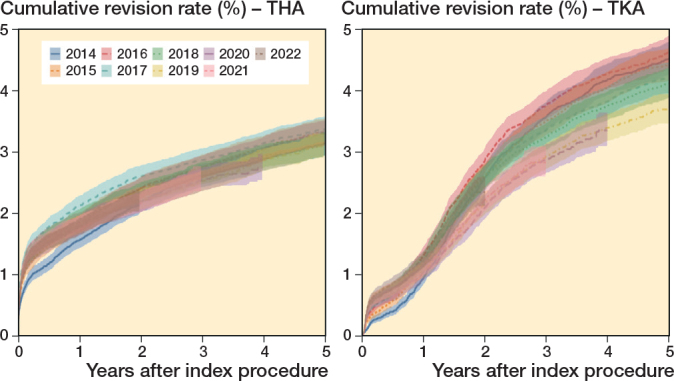
Cumulative revision rates of primary total hip and knee arthroplasties between 2014 and 2020, stratified by total hip arthroplasty (left panel) and total knee arthroplasty (right panel).

*In the third scenario,* 1-year overall revision rates were lowest in 2014 for both primary THAs (1.56%, CI 1.42–1.71) and TKAs (0.96%, CI 0.84–1.09), and highest in 2017 for THAs (2.12%, CI 1.97–2.29) and in 2016 for TKAs (1.33%, CI 1.19–1.48; Figure 4). In 2022, 1-year overall revision rates were 1.77% (CI 1.64–1.91) for THAs and 1.25% (CI 1.13–1.39) for TKAs. For the 36,637 primary THAs in 2023, the 1-year revision rate of 2022 resulted in 77 more THA revisions within 1 year compared with 2014, and 128 fewer THA revisions within 1 year compared with 2017. For the 28,249 primary TKAs in 2023, the 1-year revision rate in 2022 led to 82 more TKA revisions within 1 year compared with 2014, and to 23 fewer TKA revisions within 1 year compared with 2016. This corresponds to a reduction of 1.5% in THA revisions and 0.3% in TKA revisions, compared with the calendar year with the highest revision rates. The potential cost savings ranged from €1,920,000 to €3,200,000 for THA revisions and €345,000 to €575,000 for TKA revisions, depending on revision costs.

## Discussion

Our study presents a case analysis of the value for money of a national arthroplasty registry, which is underexplored in the literature due to the challenges of developing a rigorous study design. We found that 119 to 145 THA revisions and 106 to 134 TKA revisions needed to be prevented in the current patient population over 30 years for the benefits to outweigh the LROI costs between 2014 and 2022.

Each Dutch hospital should prevent 1.2 to 1.5 THA revisions and 1.1 to 1.3 TKA revisions in their current patient population over 30 years to outweigh the LROI costs between 2014 and 2022. The LROI, in collaboration with the Netherlands Orthopaedic Association, has implemented various quality improvement measures that may contribute to revision reduction in hospitals. In 2013, an evidence-based classification system for well-performing THAs was developed, defined as THAs with a 10-year overall revision rate of ≤ 10% or a 5-year overall revision rate of ≤ 5% [17]. After implementation of this evidence-based classification system, an increased use of proven well-performing THAs was observed, indicating that patient care and safety had improved. Later, the Orthopaedic Data Evaluation Panel classification was adopted for hip, knee, and shoulder arthroplasties to provide information on long-term results of specific prostheses. These measures significantly reduced the use of implants in the Netherlands with unknown or suboptimal long-term survival. In 2017, the LROI initiated an outlier procedure to detect prostheses and hospitals with higher revision rates than expected using funnel plots [18]. Outliers are defined as prostheses or hospitals with revision rates higher than the 99% control limits of the funnel plot. Possible outlier hospitals and manufactures of possible outlier prostheses are notified and are requested to provide an explanation and sometimes improvement strategies. If necessary, suitable measures will be taken, such as the withdrawal of an underperforming prosthesis, leading to fewer patients receiving protheses with higher revision rates than expected [19]. Moreover, the LROI promotes quality improvement by offering hospitals and surgeons real-time, detailed performance insights via dashboards and quality reports, allowing comparisons with national data and highlighting factors that may influence performance, such as prosthesis characteristics, the operating surgeon, and revision details (e.g., type and reason). This can help identify possible improvement strategies to optimize orthopedic care. Considering that 93% of hospitals performed more than 500 primary THAs and 88% more than 500 primary TKAs between 2014 and 2022, preventing fewer than 3 THA and TKA revisions over 30 years in their current patient population appears feasible, outweighing the LROI costs between 2014 and 2022.

Improving 1-year overall revision rates to the upper 99% control limit in outlier hospitals could potentially prevent 306 THA revisions and 140 TKA revisions between 2014 and 2022, which would be sufficient to outweigh the LROI costs. The additional cost savings could be used to train outlier hospitals to improve revision rates and other patient outcomes. A cluster-randomized controlled trial nested in the LROI assessed the effectiveness of a quality improvement intervention on patient outcomes after THA and TKA [20]. 10 interventional hospitals received monthly feedback on patient outcomes based on LROI data, education on data interpretation, and an action toolbox with evidence-based quality improvement initiatives based on the feedback, while control hospitals continued usual care. Patients in interventional hospitals were more likely (OR 1.24, CI 1.05–1.48) to achieve better outcomes, defined as the absence of 1-year revision, 30-day readmission, 30-day complications, and prolonged hospital stay, than those in control hospitals [20]. This suggests that registry-based quality improvement strategies enhance patient outcomes, contributing to lower healthcare costs.

Applying 2022 1-year revision rates to 2023 procedure volumes suggested reductions of 1.5% for THAs and 0.3% for TKAs compared with the calendar years showing the highest revision rates. While these reductions appear modest, they correspond to substantial associated cost savings, highlighting the potential impact of small changes in national revision rates. Although causality cannot be established, these findings provide a quantitative indication of the possible influence of registry-based quality initiatives and ongoing surveillance on revision burden.

Developing a rigorous study design to evaluate the economic impact of national arthroplasty registries is complex, as the availability of suitable data to create a control group for the registry is often lacking [7]. Study designs for conducting economic evaluations of clinical quality registries (CQR), such as national arthroplasty registries, often include trial-based and before–after designs [7,21]. In trial-based designs, the control arm may be hypothetical, assuming no registry exists, making it difficult to establish a reliable control group. Before–after designs include comparisons with pre-registry periods and are susceptible to variations in clinical practice and patient populations before and after the implementation of the CQRs. Shifts in patient demographics, such as the increasing number of younger and more vulnerable older patients undergoing THA or TKA, may contribute to higher revision rates. Conversely, advancements in surgical protocols over time could reduce revision rates. These factors highlight the difficulty of isolating the registry’s impact on revision trends and associated cost savings, as changes cannot be directly attributed to the registry [7,9]. The lack of a comparator for the CQRs limits the degree of certainty around the actual economic impact of CQRs [7].

### Limitations

PROM response rates are relatively low in the LROI and vary by hospital [11]. In this study, the available EQ-5D index scores ranged from 6.7% to 30% for primary and revision THA and TKA patients. This could affect generalizability and introduce non-responder bias. PROM responders may be more likely to be either satisfied or dissatisfied with the procedure than non-responders, potentially overestimating or underestimating the impact of a revision on quality of life and, consequently, the number of revisions that need to be prevented.

This study examined the value for money of the LROI by relating the costs received from health insurers to potential benefits from preventing revisions. However, establishment costs of the LROI were not considered in this study, leading to an underestimation of the number of revisions that need to be prevented to outweigh the total LROI costs. In contrast, the societal costs of a revision were also not considered. Patients may be unable to work for extended periods or may rely on family members, leading to economic losses and caregiver burden. This may overestimate the number of revisions that need to be prevented.

Furthermore, this study focused on the potential reduction in revisions, while this is only one benefit of national arthroplasty registries. Another benefit is the generation of data that can be used for research. Research using registry data is usually less costly than clinical trials, as data has already been collected as part of standard care. For instance, the Norwegian Arthroplasty Register observed inferior short-term outcomes with Boneloc cement compared with other types of cement, which could not be explained by patient or procedure characteristics, contributing to the withdrawal of this bone cement [22,23]. Moreover, several arthroplasty registries identified higher revision rates for prostheses with metal-on-metal bearing surfaces, leading to their near-complete worldwide withdrawal [24]. These findings could substantially reduce revisions, making a registry cost-effective for an extended period. Potential benefits emerging from research publications were not included, as those values are difficult to quantify. In addition, prevented revisions also lead to prevented re-revisions, which are even more expensive. The potential benefits from prevented re-revisions were not considered in this study. These factors contribute to an underestimation of the added value of the LROI.

### Conclusion

Preventing 119–145 THA revisions and 106–134 TKA revisions in the current patient population over 30 years would be sufficient to outweigh the LROI’s registration costs between 2014 and 2022. This corresponds to preventing about 1–2 revisions per hospital, which appears feasible. Improvements in outlier hospitals alone could already surpass this threshold. Although causality cannot be established, our findings suggest that national arthroplasty registries can provide value for money when their data is actively used to reduce revision rates. As revision prevention represents only one benefit, the true societal value of the registry is likely higher.

## Notes

MHWvV, GH, BWS, JPCG: concept and design. MHWvV, GH, JPCG: data analysis and interpretation. MHWvV: manuscript preparation. MHWvV, GH, BWS, JPCG: manuscript editing. MHWvV, GH, BWS, JPCG: manuscript review. MHWvV, GH, BWS, JPCG: final approval of the version submitted.

Handling co-editors: Li Felländer-Tsai and Philippe Wagner

*Acta* thanks Maziar Mohaddes and Petri Virolainen for help with peer review of this manuscript.

## Appendix

### Patient-level state-transition model

Patients could not transition from “Primary THA or TKA” to “> 1 year after revision”, from a revision state back to “Primary THA or TKA,” from “> 1 year after revision” to “First year after revision,” or remain in “First year after revision.” The mortality probability was based on publicly available data from Statistics Netherlands for ages 69–98, as 69 was the mean age in the study population [25]. The revision probability for both revision states was considered to be 0.015 in the first cycle, based on the 1-year revision rate of 1.5% in the LROI, as determined by Kaplan–Meier survival analysis. For the subsequent cycles, conditional probabilities were used, based on the time from the patient’s age at the primary procedure to the patient’s age at the revision. Conditional probabilities were determined using Kaplan–Meier survival analyses. Conditional probabilities of ages 70–98 was used for the remaining 29 cycles. QALYs were calculated based on the mean 12-month postoperative EQ-5D index score for both the primary and revision states. The 12-month postoperative EQ-5D index scores were obtained from the included sub-cohorts of patients with available data. The reimbursement rate for each prevented THA or TKA revision was taken into account, including the €20 not received for prevented revisions.

### Comparator definitions across analyses

#### Overall decision framework and comparator

The economic evaluation compares 2 strategies, i.e., (i) current practice with the LROI in place, in which the registry is assumed to contribute to quality improvement and thereby reduce the number of THA and TKA revisions, and (ii) a hypothetical scenario without the LROI, in which no registry-related prevention of revisions occurs and revision rates are assumed to remain unchanged.

#### Comparator in the threshold analyses

For the threshold analyses, the comparator is a 0% reduction in revision rates (i.e., no effect of the LROI on revisions). The analyses estimate the minimum percentage (and absolute number) of THA and TKA revisions that would need to be prevented for the iNMB to become positive, thereby identifying the point at which the LROI provides value for money relative to the no-registry counterfactual.

#### Comparator in the scenario analyses

*Scenario 1 (minimum reduction per hospital).* The comparator is the same as in the threshold analyses mentioned above, but translated to the hospital level. The analysis assesses whether the total number of prevented revisions required in the threshold analyses is plausible when distributed evenly across all hospitals performing THAs or TKAs.

*Scenario 2 (outlier hospitals).* The comparator is the observed performance of outlier hospitals relative to a benchmark defined by the upper 99% control limit of the national distribution of risk-adjusted 1-year revision rates. The baseline scenario assumes that outlier hospitals do not improve, whereas the intervention scenario assumes improvement up to this benchmark, consistent with a plausible mechanism through which registry feedback may reduce revisions.

*Scenario 3 (temporal changes in revision rates).* The comparator is a hypothetical situation in which 1-year revision rates remain at historically higher (or lower) levels observed in earlier calendar years. The observed revision rates in 2022 are compared against these historical extremes to approximate the potential impact of system-wide improvements over time that may be facilitated by registry-based monitoring and feedback.
